# Review: therapeutic approaches for circadian modulation of the glioma microenvironment

**DOI:** 10.3389/fonc.2023.1295030

**Published:** 2023-12-20

**Authors:** Ella A. Nettnin, Thien Nguyen, Sophia Arana, Maria Isabel Barros Guinle, Cesar A. Garcia, Erin M. Gibson, Laura M. Prolo

**Affiliations:** ^1^Department of Neurosurgery, Stanford University School of Medicine, Stanford, CA, United States; ^2^Division of Pediatric Hematology/Oncology, Lucile Packard Children’s Hospital, Palo Alto, CA, United States; ^3^Department of Psychiatry and Behavioral Sciences, Stanford University, Stanford, CA, United States; ^4^Division of Pediatric Neurosurgery, Lucile Packard Children’s Hospital, Palo Alto, CA, United States

**Keywords:** circadian, tumor microenvironment (TME), chronotherapy, glioma, glioblastoma, pediatric high-grade glioma

## Abstract

High-grade gliomas are malignant brain tumors that are characteristically hard to treat because of their nature; they grow quickly and invasively through the brain tissue and develop chemoradiation resistance in adults. There is also a distinct lack of targeted treatment options in the pediatric population for this tumor type to date. Several approaches to overcome therapeutic resistance have been explored, including targeted therapy to growth pathways (ie. EGFR and VEGF inhibitors), epigenetic modulators, and immunotherapies such as Chimeric Antigen Receptor T-cell and vaccine therapies. One new promising approach relies on the timing of chemotherapy administration based on intrinsic circadian rhythms. Recent work in glioblastoma has demonstrated temporal variations in chemosensitivity and, thus, improved survival based on treatment time of day. This may be due to intrinsic rhythms of the glioma cells, permeability of the blood brain barrier to chemotherapy agents, the tumor immune microenvironment, or another unknown mechanism. We review the literature to discuss chronotherapeutic approaches to high-grade glioma treatment, circadian regulation of the immune system and tumor microenvironment in gliomas. We further discuss how these two areas may be combined to temporally regulate and/or improve the effectiveness of immunotherapies.

## 1 Introduction

Circadian rhythms are endogenous behavioral, physiological, and molecular rhythms that follow an approximately 24-hour cycle (1). This cycle involves transcriptional-translational negative feedback loops that are regulated by the core clock components; these include brain and muscle ARN‐t like protein 1 (*BMAL1*), circadian locomotor output cycles kaput (*CLOCK)*, period proteins *(PER1, PER2, PER3)*, and cryptochrome proteins (*CRY1, CRY2)* (2). These clock components form a positive limb, which activates the expression of downstream circadian genes, and a negative limb, which dampens expression of downstream circadian genes (3). Together, BMAL1 and CLOCK form a heterodimer that comprises the positive limb of the circadian clock (4). This BMAL1-CLOCK heterodimer drives transcription of downstream genes including the PER and CRY genes, which comprise the negative limb of the clock and feed back into the nucleus to suppress their own transcription (4). Other components of the clock include REV-ERBα, which inhibits BMAL1 transcription and thus regulates the positive limb of the circadian clock (5).

These circadian genes regulate an array of physiological processes throughout the body and can influence immune system functioning, cell cycle, metabolism, apoptosis, DNA repair, and epithelial-mesenchymal transition (EMT) (6–8). Notably, several of the pathways regulated by the circadian system overlap with oncologic mechanisms of survival; therefore, alteration of the circadian clock may be instrumental in modulating survival and progression of these various cancers. The circadian clock’s regulation of such widespread processes has led to the emerging strategy of chronotherapy (9). This method involves timing the administration of treatments to a patient’s circadian rhythm to maximize benefit and minimize adverse effects (9). Understanding circadian regulation of oncologic mechanisms of survival, and how to use this knowledge to enhance treatment efficacy, will be a crucial step in advancing the treatment of cancer.

Gliomas are the most common primary brain tumor, with high-grade gliomas or glioblastoma being the most common primary malignant brain tumor in adults (10). Prognosis for glioblastoma remains poor, with a median survival of 15 months, and most patients do not survive beyond two years (10, 11). There is evidence that high-grade gliomas express circadian genes that follow diurnal rhythms (12) and appear to have deregulated expression levels compared to low-grade gliomas and surrounding non-glioma brain tissue (13–17). Expression of PER1 and PER2—genes involved in the negative limb of the circadian clock—is found to be lower in glioma cells compared to the surrounding non-cancer tissue (15). Similarly, studies have shown decreased expression of CRY1 and CRY2 in glioma cells (16). Expression of CLOCK, on the other hand, is found to be upregulated in high-grade glioma compared to low-grade glioma and non-cancerous cells (14, 18). Thus, research in gliomas has shown downregulation of circadian genes in the negative limb and an upregulation of genes in the positive limb. Experimental downregulation of CLOCK and BMAL1 in glioma stem cells (GSCs) results in cell cycle arrest and apoptosis, suggesting that this circadian deregulation is crucial to the growth of GSCs (12). In this review, we highlight the role of the circadian clock in gliomagenesis and explore how this relationship can be exploited therapeutically. We reviewed the literature for chronotherapeutic approaches to treatment, and we focus on the interplay of glioma regulation and the tumor microenvironment (TME).

## 2 Chronotherapeutic approach to high grade glioma treatment

Outcomes for patients with high-grade gliomas (grade III and grade IV glioblastoma) remain poor despite decades of clinical trials, highlighting the need for enhanced treatment efficacy (19). Recent studies showing circadian regulation of glioma pathogenesis suggest that administering treatment based on a patient’s circadian rhythms, or chronotherapy, may be a promising treatment strategy (9, 20–28). Studies exploring chronotherapeutic approaches to glioma treatment are summarized in **Table 1**.

**Table 1:** Summary of treatments and clinical outcomes for circadian regulation of gliomas.

				Sample	Patient Demographics: N (% Caucasian) Mean age Female (%)	Circadian Effect on Treatment Efficacy	Circadian Effect on Treatment Side Effects
**Conventional Treatments**	**TMZ**		Damato et al., 2021 (20)	Patients with glioblastoma	N = 166 (96.4% Caucasian) Mean age = 60.1 years Female (%) = 36.75	Morning TMZ increased OS compared to evening (1.43, 1.12–1.92 vs 1.13, 0.84–1.58 years). Effect increased to 6 months longer survival for MGMT-methylated patients treated in the morning.	N/A
			Damato et al., 2022 (21)	Patients with WHO grade III and IV or high-risk WHO grade II gliomas	N = 35 (91% Caucasian) Mean age = 56.31 years Female (%) = 43	No difference in survival was observed between AM- and PM-treated patients.	No difference in adverse events between AM- and PM-treated patients
			Slat et al., 2017 (22)	Mesenchymal glioblastoma astrocytes in mice	N/A	Maximum TMZ sensitivity occurred near the daily peak in BMAL1 expression.	N/A
			Chai et al., 2022 (23)	Patients with glioma (TCGA and CGGA databases)	N = 1,589 Female (%) = 42	Optimal TMZ administration should be at peak BMAL1 expression, which occurs at night.	N/A
	**Radiotherapy**		Zhanfeng et al., 2015 (24)	Glioma tissue in rats	N/A	Radiation delivered at peak PER2 expression led to increased apoptosis and decreased proliferation compared to trough PER2 expression.	N/A
			Zhu, Wang, Hu, & Wang, 2019 (25)	Human glioma cells (U343 glioma cell line)	N/A	High expression of PER1 was associated with increased sensitivity to radiation.	N/A
			Sapienza et al., 2021 (26)	Patients with high-grade glioma	N = 109 Mean age = 62.6 years Female (%) = 43	Treatment time (morning vs afternoon) did not affect survival outcomes.	Treatment time (morning vs afternoon) did not affect adverse events.
**Novel Treatments**	**P38 MAPK inhibitors**		Goldsmith et al., 2018 (27)	Rat glioma cells (IM3)	N/A	Glioma invasiveness is inhibited when p38 MAPK inhibitor is applied at the nadir of phosphorylated p38 MAPK expression. Invasiveness is not affected when treatment is applied at peak phosphorylated MAPK expression.	N/A
	**1A-116**		Trebucq et al., 2021 (28)	Human glioma cells (LN229)	N/A	Survival was increased when mice were treated with 1A-116 at ZT12 compared to ZT3 and vehicle conditions.	N/A

### 2.1 Conventional treatments

The standard treatment for high-grade gliomas follows the 2005 Stupp protocol which is resection followed by concurrent chemotherapy with temozolomide and radiation followed by maintenance chemotherapy (11, 29, 30). Treatment for low-grade gliomas consists of resection when amenable followed by surveillance; for unresectable tumors, biopsy followed by targeted therapy (ie. BRAF or MEK inhibitors) for progressive tumors (30–32). In this section, we review the literature on chronotherapy in these conventional glioma treatments, with a specific focus on high-grade gliomas. The efficacy and side effects profile of these treatments may be regulated by circadian rhythms in gene expression, highlighting the importance of exploring chronotherapeutic approaches to augment standard glioma treatment strategies.

#### 2.1.1 Temozolomide

Standard treatment for glioblastoma involves the DNA-alkylating agent TMZ, which acts by methylating DNA at the O6-guanine residue site (33). By methylating the DNA, TMZ induces DNA cross-linking and eventually cell apoptosis (33). Methylguanine methyltransferase (MGMT), a DNA repair enzyme, can remove the O6-methylguanine thus conferring resistance to TMZ. A subset of glioblastoma, however, express methylated MGMT which makes the repair enzyme inactive and leads to tumor cell TMZ sensitivity and prolongs patient survival (33). Recent work has suggested that adult gliomas demonstrate differential responses to TMZ depending on time of administration, suggesting a beneficial role for a chronotherapeutic approach to TMZ administration (20, 22, 23). One retrospective study on adult glioblastoma found that patients who received TMZ in the mornings had a 3.6 month longer overall survival than patients who received TMZ in the evenings (20). On further risk stratification, this survival benefit was extended to a 6 month increased overall survival in patients with O6-methylguanine-DNA methyltransferase (MGMT)-methylated glioblastoma, who received TMZ in the morning compared to MGMT-methylated patients treated in the evening (20). Given these findings, a follow-up phase II clinical trial was performed and demonstrated that chronotherapy with TMZ is feasible (21). Although this study found no difference in overall survival or adverse effects between patients treated with TMZ in the morning versus evening, the authors note that their small sample size and heterogenous patient population limit what can be concluded in terms of survival benefit (21). In summary, increased TMZ sensitivity in the AM may be driven by diurnal and differential expression of MGMT (34, 35) and may be a suitable target for larger studies in the future.

Another proposed mechanism of differential efficacy of TMZ in gliomas may relate to direct interaction of TMZ to circadian gene expression. One hypothesis is that TMZ sensitivity is specifically tied to the cyclic expression of BMAL1, the binding partner of CLOCK that helps drive transcription of downstream circadian genes (PERs and CRYs) and an essential part of the circadian clock (36, 37). Investigators used both primary human glioblastoma cells and primary mesenchymal murine glioblastoma astrocytes to show that cells are most sensitive to TMZ when it is administered near the daily peak in BMAL1 expression (22). This temporal effect disappeared after a CRISPR-mediated loss of BMAL1, suggesting that the chronotherapeutic sensitivity of TMZ is dependent on BMAL1 (22). Another study took a bioinformatics approach utilizing a database with information on drug sensitivity and gene expression profiles, demonstrating that higher expression of BMAL1 is significantly correlated to higher TMZ sensitivity (23). These findings link the TMZ chronotherapeutic outcomes to the molecular components of the circadian clock. Our understanding of chronotherapy for TMZ remains limited by the lack of large, prospective randomized control trials and future studies should explore how to best time TMZ administration to maximize benefit (38). One promising approach could involve the use of high-throughput sequencing modalities to delineate transient changes in circadian gene expression, which would allow for more accurate dosing of TMZ therapy. RNA sequencing analysis has previously been used to identify molecular pathways involved in TMZ resistance in glioblastoma and could similarly be used to explore circadian regulation of TMZ sensitivity (39, 40). By employing high-frequency output, clinicians may be able to appropriately time treatment to the tumor circadian rhythms in their patients.

#### 2.1.2 Radiotherapy

Unlike TMZ, chronotherapeutic strategies for radiation therapy treatment of gliomas remain controversial. Broadly, the expression of circadian genes *Per1* and *Per2* are thought to modulate the efficacy of radiotherapy in the treatment of gliomas (24, 25). Per1 expression levels are found to modulate the transcription of a variety of genes, including *p53* target genes and checkpoint components for DNA repair (25). Decreasing PER1 expression also results in decreased expression of the *Chk2-P53* signaling pathway and C-Myc, which are integral components of DNA-damage repair and apoptosis, respectively (25). As a result, PER1 is seen to play an important role in regulating the DNA damage response and subsequent apoptosis caused by radiation (25). Zhu et al. demonstrated that downregulating PER1 in human glioblastoma cells *in vitro* using an shRNA lentivirus resulted in minor DNA damage and reduced apoptosis in response to radiation compared to controls (25). Similarly, Zhanfeng et al. found this same positive correlation *in vivo*, showing that high expression of PER2 in glioma murine tissue was associated with an increased sensitivity to radiation. Specifically, there were higher levels of apoptosis and lower levels of proliferation when radiation was delivered at peak PER2 levels versus trough PER2 levels (24). This suggests there is a benefit of optimizing timing of radiotherapy based on circadian cycling (24). However, the survival benefits seen *in vitro* in U343 glioma cells and *in vivo* in glioma-bearing rats were not found in humans. In a retrospective study of 109 patients with high-grade gliomas, Sapienza et al. found no significant difference in progression-free survival or overall survival between patients treated with radiotherapy in the morning versus the afternoon (26). This may be because: i) other mechanisms overpower a modest survival benefit of radiation timing in humans, ii) that the time of radiation effect on tumor cells is much longer than what would occur within a circadian cycle, iii) there are other confounders such as concordant steroid administration that may reset the circadian clock, or iv) that there is not a measurable effect in humans. Despite this finding, the outcomes and toxicity-related benefits of chronoradiotherapy have been shown in other cancers (41–43). For example, a retrospective review on patients with rectal cancer found that patients who received radiotherapy after 12:00 pm had improved response rates compared to patients who were primarily treated before 12:00 pm (43). Similar benefits to chronoradiotherapy were shown for prostate cancer (42) and for bone metastases (41). These conflicting results suggest the need for further exploration into the potential benefits of chronotherapeutic approaches to radiation therapy in malignant brain tumors. As mentioned above, modern sequencing technologies may prove useful in elucidating the role for chronotherapeutic approaches to radiotherapy. Past studies have utilized total RNA sequencing to understand mRNA expression changes underlying radiotherapy resistance in glioblastoma (44), thus a similar analysis of circadian gene expression may allow for effective chronotherapeutic radiation therapy.

### 2.2 Novel treatments

Given the poor outcomes with the standard high-grade glioma treatment of surgical resection, chemotherapy, and radiotherapy, there is an urgent need for new therapeutic strategies. To further optimize novel therapies, we discuss the potential of chronotherapeutic approaches to augment new promising treatments in high-grade gliomas.

#### 2.2.1 Immunotherapy

The development of immunotherapies, such as chimeric antigen receptor (CAR) T-cells, was a promising advancement in treatment for high-grade gliomas, yet survival outcomes remain poor with immunotherapy. Barriers to immunotherapies such as CAR T-cell therapy in gliomas include off-target effects, poor infiltration into the tumor, and an immunosuppressive tumor microenvironment (45). CAR T-cell response can be enhanced by the addition of a synthetic agonist for the circadian gene Retinoid-related orphan receptor γ (*RORγ*) (46, 47). Hu et al. demonstrate that adding RORγ, a circadian regulator of BMAL1 (48), to melanoma tumor-specific T cells *in vitro* and transferring them to tumor-bearing mice results in reduced tumor growth and improved T cell survival (47). Similarly, in a separate study, they show that adding RORγ during *ex vivo* expansion of a patient’s immune cells increases the antitumor activity of T-helper 17 (Th17) cells that are engineered with a CAR (46). This antitumor activity is seen to persist long-term, with elevated levels of cytokines detected months after infusion (46). The antitumor activity of RORγ involves increasing production of cytokines such as Interleukin-17 and Granulocyte macrophage colony-stimulating factor, as well as co-stimulatory receptors tumor necrosis factor receptor superfamily member 9 (CD137) and Cluster of Differentiation (CD) 226 (47). RORγ also decreases immunosuppression by attenuating the activity of regulatory T cells (Tregs) and reducing expression of CD39, CD73, Programmed Cell Death Protein 1 (PD-1), and T cell immunoreceptor with Ig and ITIM domains (TIGIT) (47). These results offer promising evidence that manipulating the circadian clock may enhance the efficacy of CAR T-cell treatment. Future research should examine whether these findings extend to high-grade gliomas and whether time of administration affects the efficacy and side effect profile of CAR T-cell therapy.

#### 2.2.2 Small molecule inhibitors

Several small molecule inhibitors have limited efficacy in the treatment of gliomas, despite targeting key growth pathways. Given their molecular potential, their response may be enhanced through further regulation of the tumor’s circadian rhythm. These small molecular inhibitors target the epidermal growth factor (EGF), and mitogen-activated protein kinase (MAPK) pathways and may have maximal therapeutic effect by understanding the chronoregulation of gliomas.

##### 2.2.2.1 Epidermal growth factor receptor inhibitors

EGFR is one of the most common mutation sites in glioblastoma, making it an important therapeutic target (49). Mutations in the glioblastoma EGFR are primarily gene amplifications, suggesting the use of EGFR inhibitors as a potential treatment (49). Despite this promise, EGFR inhibitors have limited efficacy in treating glioblastoma (50). Recent work in mice has demonstrated circadian control of EGFR signaling, with EGFR signals being low during the active phase and high during the resting phase (51, 52). Lauriola et al. used xenograft athymic nude mouse models injected subcutaneously with N87 human gastric cancer cells to further show that administration of an EGFR inhibitor drug during the resting phase, when EGFR levels were elevated, resulted in a lower tumor volume compared to administration during the active phase (51). Advances in sequencing technologies have elucidated correlations between circadian genes and clinically actionable genes such as EGFR (53), further paving the way for more personalized chronotherapeutic approaches to treatment. Together, these results suggest that chronotherapy may be key in successfully applying EGFR inhibitors as a treatment for high-grade gliomas.

##### 2.2.2.2 p38 MAPK inhibitors

Inhibitors of p38 MAPK have garnered attention as potential therapeutic agents given the correlation between high activity of p38 MAPK and poor prognosis in cancers, such as glioblastoma (3, 54). The p38 MAPK pathway is a signaling pathway known to play an important role in various cell processes including apoptosis, proliferation, and differentiation (27, 54). Increased p38 MAPK activity is associated with decreased apoptosis and less sensitivity to TMZ, suggesting that p38 MAPK inhibitors may sensitize tumors to chemotherapy (54). The low efficacy and high rate of off-target effects, however, limit the current potential of these inhibitors (55). Recently, there has been evidence of circadian regulation of the p38 MAPK activity, therefore highlighting a connection between the p38 MAPK pathway and the circadian clock (56–58). One study demonstrated this circadian regulation of p38 MAPK activity *in vitro* using murine glioma cells (27). The authors showed that p38 MAPK levels are cyclic in human astroglia, but remain elevated and arrhythmic in murine glioma cells (27). By inhibiting p38 MAPK in glioma cells at a time of day when levels are normally low in the human astroglia, there is a significant reduction in glioma invasiveness (27). These findings suggest that the therapeutic use of p38 MAPK inhibitors for high-grade glioma could be improved by timing administration of the drugs to complement the circadian rhythms in p38 MAPK activity.

##### 2.2.2.3 1A-116

The novel drug 1A-116 was recently proposed as a potential treatment for tumors such as glioblastoma (59–61). This drug is a small molecule that reduces Rac1 activation levels by inhibiting interactions between Rac1 and guanine nucleotide exchange factors (GEFs) (60). These Rac1-GEF interactions are crucial for fundamental cellular processes such as proliferation, migration, cytoskeletal reorganization, and apoptosis (60). As a result of inhibiting Rac1 activation, 1A-116 is seen to effectively reduce tumor progression in a variety of cancers including gliomas (59, 61). One study demonstrated circadian regulation of 1A-116 efficacy, showing increased survival time for mice treated with the drug at the end of the light period compared to those treated at the beginning of the light period (28). The study also found that administration of 1A-116 at the time when PER1 levels are high and BMAL1 levels are low resulted in the strongest effects on cell proliferation, apoptosis, and migration (28). These temporal responses disappeared upon knocking down BMAL1, suggesting a dependence of 1A-116 sensitivity on circadian regulation (28). Mechanisms driving these time-dependent effects may include cyclic expression of Rac1, circadian regulation of downstream components in the affected pathways, and circadian regulation of 1A-116 entry into tumor cells (28). Together, these results offer promising evidence for novel glioma treatments, which can have an enhanced benefit when administration is timed to circadian rhythms.

## 3 Circadian modification of the tumor microenvironment

The heterogenous and ever-changing nature of high-grade gliomas are due, in part, to their ability to modify the tumor microenvironment (TME) to overcome selection pressures from the outside environment (62). The TME refers to the non-cancerous cells in the tumor including fibroblasts, endothelial cells, neurons, and immune cells, as well as non-cellular components such as the extracellular matrix, cytokines, and growth factors (63). There is a reciprocal relationship between cancer cells and the TME, and the dynamic nature of the glioma TME contributes to the low efficacy of various treatments, including immunotherapies, by creating an immunosuppressive environment (62, 63). Circadian clock components regulate the TME through effects on angiogenesis, inflammation, and immune suppression, thus influencing the TME’s role in tumor progression (62, 63). Targeting circadian regulation of the TME therefore offers a promising therapeutic strategy by interfering with glioma pathogenesis.

### 3.1 Extracellular microenvironment

**Figure 1:**
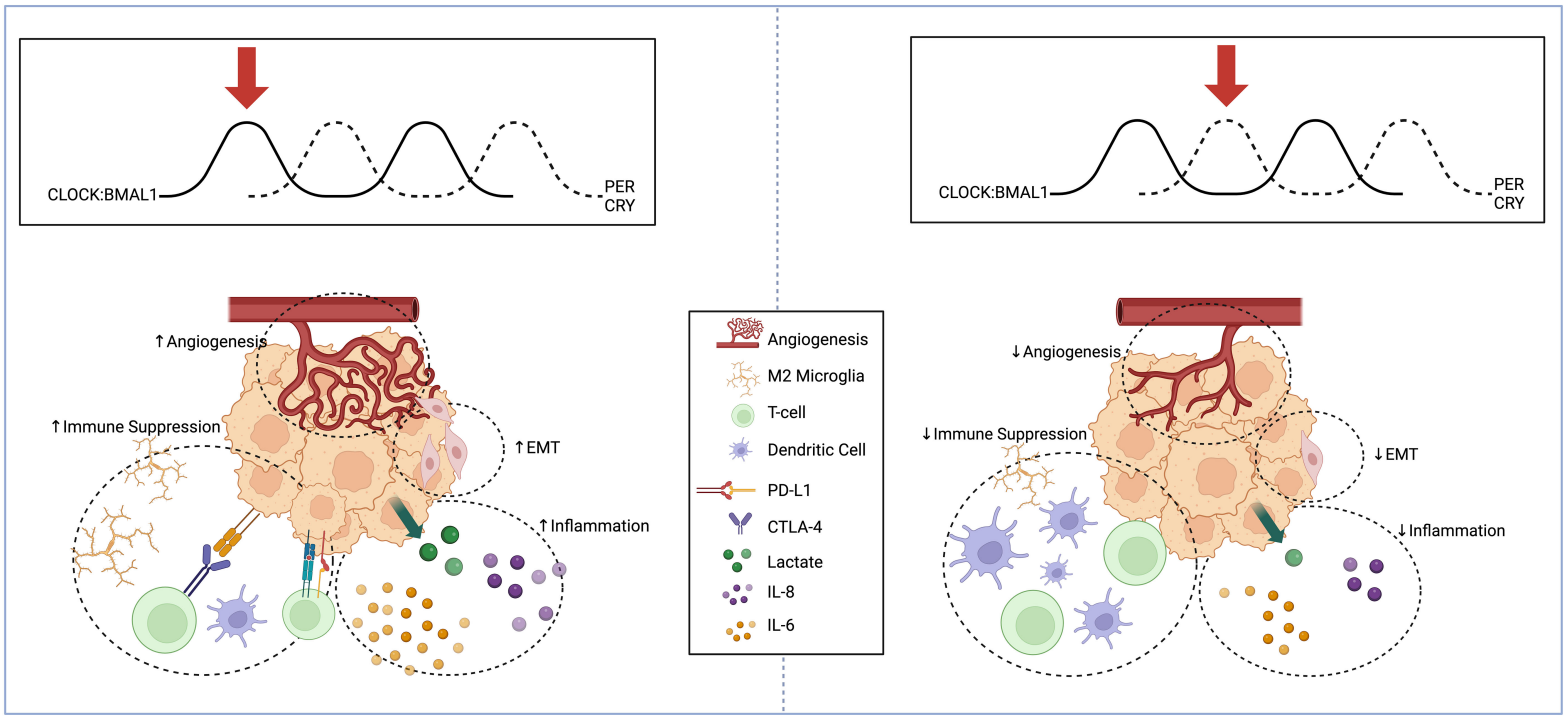
Schema displaying circadian regulation of high-grade glioma extracellular microenvironment at two different points in the circadian cycle. High-grade glioma microenvironment represented at two different points in the circadian cycle. When BMAL1 and CLOCK levels are high and PER and CRY levels are low (Left), there is an increase in pro-angiogenic factors, mesenchymal differentiation, inflammatory cytokine release, and immunosuppressive microglia. When BMAL1 and CLOCK levels are low and PER and CRY levels are high (Right), there is reduced angiogenesis, suppression of EMT, reduced inflammatory markers, and reduced recruitment of immunosuppressive microglia and expression of PD-L1. Created with BioRender.com.

In this section, we summarize the literature on circadian regulation of the glioma TME, focusing on the adaptive measures and changes in the tumor’s surroundings (**Figure 1**). The non-cancerous cells and environmental components that comprise the TME include endothelial cells, immune cells, cytokines, and the extracellular matrix (63). We discuss circadian regulation of angiogenesis, EMT, and immune targets, all critical to glioma proliferation and invasion.

#### 3.1.1 Angiogenesis

Gliomas can modify their TME through angiogenesis, mediated through the vascular endothelial growth factor (VEGF) pathway and resulting in the generation of new blood vessels to deliver necessary nutrients for further tumor growth and expansion (64, 65). This process is thought to involve circadian regulation, according to results of a study by Pang et al. (66). In this study, authors utilize gene set enrichment analysis on patient glioblastoma samples from publicly available databases to show that angiogenesis is one of the most enriched pathways in glioblastoma tumors with high BMAL1 expression compared to those with low BMAL1 expression (66). Additionally, they use glioblastoma patient-derived cells to show that angiogenesis is greatly reduced in glioblastoma cells with depletion of either CLOCK or BMAL1 (66). Furthermore, the administration of SR9009, a selective androgen receptor modulator that activates the circadian gene *Rev-Erbα*, decreases angiogenesis in glioblastoma *in vitro* and reduces intratumoral blood vessels *in vivo* in murine glioma models (66). Together, these findings highlight the effect of circadian regulation on angiogenesis.

**Figure 2:**
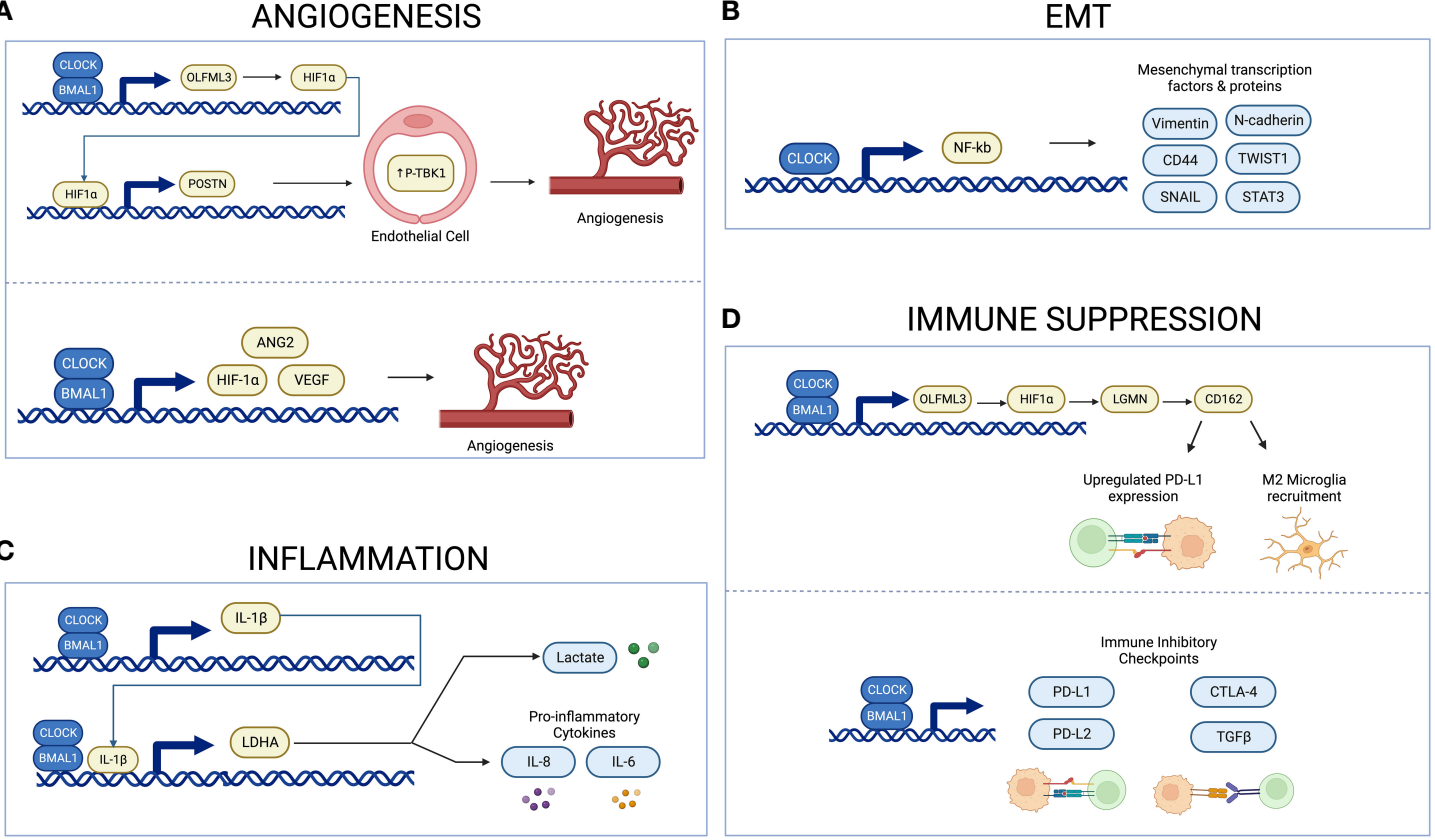
Schema illustrating circadian regulation of angiogenesis, inflammation, EMT, and immune suppression. The CLOCK-BMAL1 complex regulates expression of proangiogenic factors and microvascular density **(A)**. CLOCK upregulates NF-kB activity, thus inducing expression of mesenchymal proteins and transcription factors **(B)**. The CLOCK-BMAL1-IL-1B-LDHA axis regulates expression of lactate and proinflammatory cytokines **(C)**. The CLOCK-BMAL1 complex regulates recruitment of immunosuppressive microglia and expression of immune inhibitory checkpoints **(D)**. Created with BioRender.com.

More specifically, circadian genes regulate the expression of several angiogenic factors. For example, the Pang et al. study suggests that CLOCK and BMAL1 drive expression of the pro-angiogenic factor periostin (POSTN) in endothelial cells (66). They propose a mechanism through which the CLOCK-BMAL1 complex regulates expression of the olfactomedin like 3-hypoxia-inducible factor 1-alpha (HIF-1α) axis, which upregulates POSTN and activates TANK-binding kinase 1 signaling in endothelial cells (66). Similarly, a study by Wang et al. found that expression of BMAL1 positively correlates with microvascular density, as well as angiogenic factors HIF-1α, Angiopoietin 2 (ANG2), and VEGF in gliomas (67). Correspondingly, inhibition of BMAL1 *in vitro* using primary glioma cells results in decreased expression of HIF-1α and VEGF (67). It is likely that BMAL1 promotes angiogenesis through its modulation of these angiogenic factors. Similar results are observed in other cancer types. For example, overexpression of CLOCK in human colorectal carcinoma cell lines correlates with increased expression of angiogenesis-related genes such as *HIF-1α*, *ARNT* and *VEGF*, with CLOCK knockdown showing the opposite results (68). A study in sarcoma and melanoma showed circadian rhythms in VEGF expression are regulated by *Period* and *Cryptochrome1*, and *in vivo* chronotherapeutic administration of anti-angiogenic agents during early light phase decrease tumor growth to a greater degree than administration during early dark phase (69). Additionally, a study in zebrafish embryos showed that BMAL1 positively regulates VEGF expression while PER2 negatively regulates expression (70). Altogether, these findings elucidate the mechanism underlying circadian regulation of angiogenesis through angiogenic factors such as HIF-1α and VEGF, and they highlight the therapeutic potential of targeting this pathway (**Figure 2A**).

#### 3.1.2 Epithelial-mesenchymal transition

The epithelial-mesenchymal transition (EMT) allows glioma cells to migrate and invade into surrounding tissue (64) and circadian regulation of EMT may provide a novel target for manipulation of the glioma microenvironment to inhibit the invasive nature of high-grade gliomas. In glioblastoma tissue and in cell culture, CLOCK was found to positively regulate glioblastoma migration by upregulating activity of transcription factor nuclear factor-κB (NF-κB), thus promoting mesenchymal differentiation (18, 71). NF-κB is a ubiquitous transcription factor, and one of its roles in glioblastoma involves inducing expression of mesenchymal transcription factors and mesenchymal proteins such as CD44, vimentin, and N-cadherin (71). Additionally, a study by Yu et al. in glioblastoma cells found that knocking down the nuclear receptor REV-ERBß, a repressor of circadian genes, appears to suppress EMT and metastasis of glioblastoma cells (72). Similarly, circadian regulation of EMT has been shown in other cancers such as colorectal cancer. Specifically, increased expression of CLOCK or BMAL1 in colorectal cancers correlates with increased mesenchymal markers and decreased epithelial markers (73). Conversely, silencing CLOCK or BMAL1 has the expected opposite effect, downregulating mesenchymal markers and increasing epithelial ones (68, 73). In breast cancer cells, reduced expression of the circadian gene PER2 was found to correlate with increased expression of the pro-EMT genes Snail Family Transcriptional Repressor 2 (*SLUG*), Snail Family Transcriptional Repressor 1 (*SNAI1*), and Twist-related protein 1 (*TWIST1*) (74). These results suggest a link between the circadian system and increased EMT in various cancer models (**Figure 2B**). Future studies should determine whether this mechanism is important in glioblastoma as well.

#### 3.1.3 Inflammation

The circadian clock plays a critical role in regulating the immune system through modulation of cytokine expression such as proinflammatory cytokine interleukin-1ß (IL-1ß) (75). Inflammation in the context of cancer is often tumor-promoting by supplying growth factors, proangiogenic factors, and enzymes that modify the extracellular matrix (64). Tumor-derived lactate plays several roles in tumor development, including serving as a proinflammatory mediator that increases cytokines such as IL-1ß, and lactate dehydrogenase A (LDHA) is integral in producing lactate (75, 76). Circadian genes CLOCK and BMAL1 are found to regulate expression of IL-1ß and LDHA in gliomas, with suppression of CLOCK and BMAL1 leading to a reduction in LDHA and IL-1ß levels (75). Similarly, knocking down BMAL1 or CLOCK diminishes the IL-1ß-induced increase in lactate and proinflammatory cytokines such as IL-8 and IL-6 (75). Increased expression of each part of this CLOCK-BMAL1- IL-1ß-LDHA feedback loop is correlated with poor prognosis and shorter survival (75). These findings therefore demonstrate an autocrine signaling loop that could be targeted to disrupt the dysregulated inflammation and metabolism that benefits glioma cells (**Figure 2C**).

Glucocorticoids, such as dexamethasone, are often used to alleviate cerebral edema and inflammation from intracranial tumors (77). Recently, there have been more studies linking chronopharmacology of glucocorticoid administration and the expression of circadian genes in the immune cells (78). For example, a study by Fonken et al. showed that glucocorticoids entrain circadian clock gene expression in microglia by inducing the expression of the *Per1* gene (78). This study also showed that microglia demonstrate temporal fluctuations in inflammatory cytokines such as tumor necrosis factor α (TNFα), IL-1β and IL6 (78). These findings show that microglia demonstrate circadian rhythms in inflammatory responses, and these rhythms can be influenced by administration of glucocorticoids (78). Glucocorticoids are widely used among patients with all brain tumors, and these results suggest an important link between steroid administration and circadian-regulated changes in immune cells in the glioma TME. Future studies should examine whether there are differential effects in treatment response, based on what time of day the glucocorticoids are administered.

#### 3.1.4 Immune suppression

Recently, circadian markers such as CLOCK and BMAL1 have been tied to immune suppression in glioblastomas by increasing microglia infiltration. High CLOCK expression is positively correlated with an increase in microglia and a decrease in CD8 activated T-cells and dendritic cells (79). In addition, both CLOCK and BMAL1 expression correlate with expression of microglial markers (79) and the infiltrating microglia are biased towards the immunosuppressive (M2) phenotype (79, 80). Overall, by increasing the expression of immunosuppressive microglia in the TME, CLOCK and BMAL1 genes can effectively minimize the response of glioma to immunotherapies.

Because of their interference with circadian recruitment of microglia, these circadian genes can decrease immune suppression in glioma. For example, administration of SR9009, the REV-ERBα agonist that inhibits BMAL1 expression, decreases intratumoral immune-suppressive microglia in GSCs (80). Similarly, depletion of CLOCK or BMAL1 in murine models results in reduced infiltration of microglia and improved overall survival (79). These studies highlight circadian regulation of immune suppression in the glioma TME and demonstrate how this regulation can be targeted therapeutically.

A specific signaling pathway between GSCs and microglia, the CLOCK-olfactomedin-like 3 (OLFML3)-HIF1α-legumain (LMGN)-cluster of differentiation 162 (CD162) axis, has been identified and serves as a potential therapeutic target for glioblastoma (**Figure 2D**) (80). GSCs utilize CLOCK and BMAL1 to transcriptionally regulate LMGN and OLFML3, two chemokines that promote recruitment of microglia (79, 80). Expression of the circadian gene CLOCK specifically demonstrates a positive correlation with LMGN and OLFML3 in glioblastoma (79, 80). Inhibiting any part of this CLOCK–OLFML3–HIF1α–LGMN axis results in reduced immunosuppressive microglial recruitment and prolonged survival in glioblastoma murine models (80). Similarly, treatment with either SR9009 or anti-CD162 enhances survival in this glioblastoma mouse model in response to anti-programmed cell death protein 1 (PD1) therapy (80). This finding is significant because gliomas are known to upregulate immune checkpoint molecule programmed death-ligand 1 (PD-L1) in order to suppress the immune response and evade immunotherapies (81). Targeting the CLOCK–OLFML3–HIF1α–LGMN–CD162 axis reduces PD-L1 expression and augments anti-PD1 therapy in glioblastoma mice, overall highlighting the therapeutic potential of this pathway (80).

The circadian clock is associated with immune evasion in tumors (82). In particular, a study by Wu et al. found that the circadian clock is associated with an immune evasion phenotype (82). Using RNA-sequencing data to infer level of immune cell infiltration, they demonstrated a positive correlation of core circadian genes with infiltration of Tregs and Mast cells, but a negative correlation with infiltration of Th2 cells, Th1 cells, natural killer T cells, CD8 T cells, CD8 naïve T cells, and CD4 T cells (82). This finding shows the broad impact of the circadian clock on the immune cells that infiltrate the TME. Using bioinformatics approaches, the authors demonstrate that the disrupted circadian clock in cancer contributes to T-cell exhaustion through persistent elevation of inhibitory checkpoints (82). Specifically, there was a positive correlation between all clock genes and immune inhibitory checkpoints PD-L1, PD-L2, cytotoxic T-lymphocyte–associated antigen 4 (CTLA-4) and transforming growth factor beta (TGFB) (**Figure 2D**) (82). Treatments aimed at targeting the circadian system in gliomas may therefore diminish the ability of these tumors to evade the immune system.

### 3.2 Intracellular mechanisms

In the previous section we reviewed the role of the extracellular tumor microenvironment on circadian rhythms. Here we will discuss potential intracellular pathways that may be targeted for chronotherapy.

#### 3.2.1 MGMT-methylation

As previously mentioned, MGMT plays a critical role in promoting TMZ-resistance in gliomas by repairing double-stranded DNA breaks (33). Studies by Damato et al. demonstrate a significant 6-month difference in survival between patients treated in the AM and PM, highlighting the role of the circadian system on TMZ sensitivity in MGMT-methylated gliomas (20). Further studies by Marchenay et al. and Martineau-Pivoteau et al. demonstrate circadian rhythms in MGMT protein activity in both human and mouse cells, respectively (34, 35). Marchenay et al. use serial blood samples across a 24-hour period from healthy volunteers to demonstrate circadian rhythms in MGMT activity in circulating mononuclear cells (34). Similarly, Martineau-Pivoteau et al. use liver samples obtained from mice at eight different circadian times to demonstrate circadian rhythms in MGMT activity, with the highest activity occurring during the active period (35). These findings demonstrate circadian regulation of MGMT-methylation, and understanding this cycling may allow for more precise timing of TMZ administration to maximize overall survival.

#### 3.2.2 IDH 1/2 mutations

Mutations in isocitrate dehydrogenase 1 (IDH1) and isocitrate dehydrogenase 2 (IDH2) are associated with improved survival rates among patients with glioblastoma and are positive prognostic predictors of overall survival (83, 84). However, there are few studies examining the role of IDH mutations in tumorigenesis and in modulating the circadian cancer pathway. Although most studies exploring circadian regulation of gliomas do not stratify based on IDH mutation status, De La Cruz Minyety et al. found that PER gene expression predicts survival in high-grade glioma patients independently of IDH mutational status (84). Interestingly, another study of *in vitro* glioma cells demonstrated that IDH1 mutations are associated with lower expression of BMAL1, CLOCK, PER genes, and CRY genes compared to their wildtype counterparts (85). Together these findings suggest that IDH mutations may regulate circadian gene expression, but do not show clear evidence for circadian regulation of IDH mutational status. Given the paucity of research into this important prognostic factor for glioblastoma, further studies should elucidate whether IDH mutational status could be used as a marker for assessing the effectiveness of chronotherapy.

#### 3.2.3 Growth factor axis

High-grade gliomas are known to express growth factors and growth factor receptors (86), and several malignant behaviors such as proliferation, invasion, angiogenesis, and decreased apoptosis involve growth factor signaling (87). For example, Insulin Growth Factor 1 (IGF-1) receptor signaling is one mechanism through which GSCs become resistant to radiotherapy (88). Specifically, acute radiation increases expression of IGF1R and secretion of IGF-1, which activates the phosphatidylinositol 3-kinase (PI3K)-Protein kinase B (Akt) pathway to prevent apoptosis and promote survival (88, 89). Recent work by Alonso-Gomez et al, Mazzoccoli et al, and Chaurdhari et al. revealed IGF-1 levels in the liver and serum exhibit circadian rhythms (90–92). Additionally, circadian regulation of IGF-1 has been shown to regulate cancer progression in non-small cell lung cancer cells (90–93). IGF-1 entrains the circadian clock in the liver, highlighting the two-way relationship between circadian-regulated targets and the circadian clock itself (94–96). Similarly, nerve growth factor (NGF) was found to entrain the circadian clock in hamsters, and epidermal growth factor (EGF) is seen to induce clock gene expression in neural stem cells (97–99). Likewise, fibroblast growth factor (FGF) and platelet-derived growth factor (PDGF) induce robust PER1 expression in murine fibroblasts (100). Together, these data show that regulation of growth factors represents another bi-directional link between the circadian clock and cancer biology. This link should be further researched and targeted to potentially decrease radiotherapy resistance in glioblastoma.

## 4 Pediatric high grade gliomas

Cancer is a leading cause of death amongst children, with brain tumors, specifically, being the top cause of cancer-related death in children (101). However, there is a lack of effective treatment options for childhood brain tumors and most treatments were developed for adult cancers and applied to children despite the tumors being quite different in nature (102). We discuss the available data on circadian regulation of pediatric high-grade gliomas and how the clock may be targeted for treatment.

### 4.1 Circadian regulation of the tumor microenvironment in pediatric gliomas

Immune cells play a crucial role in the TME of both high- and low-grade pediatric gliomas (103, 104). Pediatric high-grade gliomas are enriched in genes related to microglia and macrophages, and tumor-associated macrophages are specifically found to promote the growth of low-grade pediatric gliomas (103–105). Daginakatte et al. use Neurofibromatosis-1 (NF1), a glioma predisposition syndrome, to evaluate the role of microglia in glioma growth (106). They show that NF1-heterozygous microglia demonstrate increased proliferation, motility, and genes associated with microglial activation (106). When they inhibit the microglia activity *in vivo* using optic pathway glioma mouse models, there is reduced low-grade glioma proliferation (106). The mechanism underlying microglia stimulation of pediatric glioma growth is not yet fully known; one hypothesis is that microglia release chemokines and paracrine factors, such as hyaluronidase, to increase glioma proliferation (105). These findings suggest a similar role for immune cells in the survival of pediatric high-grade gliomas, but there remains a gap in our understanding the role of these cell types play in tumor progression. Future research should explore whether there is a survival benefit in immune cell targeted treatments at different times of the circadian cycle in pediatric high-grade gliomas.

### 4.2 Chronotherapy in pediatric tumors

Although there are no studies to date on the application of chronotherapy to pediatric high-grade gliomas, studies have shown a benefit of chronotherapy in pediatric patients with acute lymphoblastic leukemia (107, 108). These studies have found increased progression free survival in pediatric patients with acute lymphoblastic leukemia when chemotherapy is administered in the evening versus the morning, with a higher risk of relapse for those being treated in the morning (107, 108). Specifically, Schmiegelow et al. found a higher probability of event free survival for patients administered oral methotrexate (MTX) and 6-mercaptopurine (6MP) in the evening versus the morning, with a median follow-up of 78 months (108). Similarly, Rivard et al. found that for patients surviving disease-free for more than 78 months, there was a greater risk of relapse for patients taking MTX and 6MP in the morning versus the evening (107). A potential benefit of timing of chemotherapy for pediatric high-grade gliomas should be explored in future studies. Future studies should also explore the use of chronotherapy for other pediatric high-grade glioma treatments, such as CAR T-cell therapy. Pediatric high-grade gliomas such as diffuse intrinsic pontine gliomas (DIPGs) are fatal, but the recent clinical success of CAR T-cell therapy in DIPGs (109) highlights a potential area for chronotherapeutic augmentation. Overall, there is a dearth of research into chronotherapy in pediatric tumors, and more research is needed in this area in order to expand treatment options and efficacies among these patients.

While the cyclic expression of circadian genes may affect the efficacy of administered drugs, the pharmacological agents also affect the circadian system. Steroids such as dexamethasone, for example, which are often administered to children with brain tumors, may affect the circadian clock. Studies on children with acute lymphocytic leukemia have shown that dexamethasone dampens circadian activity rhythms in these patients, which leads to decreasing daily trends of peak activity during dexamethasone treatment (110). This study also shows that increased fatigue is associated with the dampened circadian activity rhythms, but notes that the relationship between dexamethasone and fatigue is complex and requires further investigation (110). Similarly, a study on children with central nervous system cancers demonstrated dysregulated circadian activity rhythms as a result of chemotherapy, and circadian dysregulation is known to affect health-related outcomes including quality of life, responsiveness to chemotherapy, relapse, and fatigue (111). Further research is needed to explore the health outcomes related to circadian dysregulation and interventions that restore rhythmicity. Overall, these results demonstrate the potential use of chronotherapeutic treatment of pediatric high-grade glioma to both increase the efficacy of cancer treatment as well as decrease negative side effects of the treatments.

## 5 Conclusion

In summary, deregulation of circadian genes is thought to play a role in the pathogenesis of high-grade gliomas. Circadian regulation of the TME and the immune system may be a route through which gliomas manipulate their environment to enhance their survival. Because of the link between the circadian clock and glioma pathology, chronotherapy offers a promising adjunct to the low efficacy and high side effect profile of existing glioma treatments. Future work should explore the potential of targeting circadian regulation of the TME and timing immunotherapies to maximize their benefit and minimize side effects. Rapidly advancing technologies such as high-throughput sequencing offer the unique opportunity to dissect the heterogenous and dynamic TME and can be used to further elucidate circadian regulation of the TME. For example, these technologies can be used to understand circadian-regulated changes in TME composition and to identify predictive biomarkers related to these circadian-driven changes. Additionally, most of the present research exploring circadian regulation of gliomagenesis focuses on adult tumors. Future work should focus on circadian regulation of pediatric gliomas because of the distinct lack of research and therapies directed towards this patient population. Effectively timing treatments based on circadian regulation is a novel approach that may improve survival in pediatric high-grade gliomas. Ongoing research into the circadian regulation of TME and immune targets may result in the improved therapeutic outcomes that are urgently needed for high-grade gliomas.

## Author contributions

EN: Conceptualization, Investigation, Writing – original draft, Writing – review & editing. TN: Conceptualization, Writing – review & editing. SA: Visualization, Writing – review & editing. MB: Visualization, Writing – review & editing. CG: Writing – review & editing. EG: Writing – review & editing. LP: Conceptualization, Funding acquisition, Supervision, Writing – review & editing.
